# Seroprevalence of Human Immunodeficiency Virus, Hepatitis B Virus, Hepatitis C Virus, and *Treponema pallidum* Infections among Blood Donors on Bioko Island, Equatorial Guinea

**DOI:** 10.1371/journal.pone.0139947

**Published:** 2015-10-08

**Authors:** Dong-De Xie, Jian Li, Jiang-Tao Chen, Urbano Monsuy Eyi, Rocio Apicante Matesa, Maximo Miko Ondo Obono, Carlos Sala Ehapo, Li-Ye Yang, Hui Yang, Hui-Tian Yang, Min Lin

**Affiliations:** 1 Laboratory Medical Center, The People’s Hospital of Jiangmen, Jiangmen; The Chinese Medical Aid Team to the Republic of Equatorial Guinea, Guangzhou, Guangdong Province, People’s Republic of China; 2 Department of Infectious Diseases, Renmin Hospital; Department of Parasitology, College of Basic Medicine, Hubei University of Medicine, Shiyan, Hubei, People’s Republic of China; 3 Laboratory Medical Center, Huizhou Municipal Central Hospital, Guangdong, Huizhou, Guangdong, People’s Republic of China; 4 Central Blood Transfusion Service; Medical Laboratory, Malabo Regional Hospital, Malabo, the Republic of Equatorial Guinea; 5 Central Laboratory, Chaozhou Central Hospital, Southern Medical University, Chaozhou, Guangdong, People’s Republic of China; University of Cincinnati College of Medicine, UNITED STATES

## Abstract

**Background:**

Regular screening of transfusion-transmissible infections (TTIs), such as human immunodeficiency virus (HIV), hepatitis B and hepatitis C virus (HBV and HCV, respectively), and *Treponema pallidum*, in blood donors is essential to guaranteeing clinical transfusion safety. This study aimed to determine the seroprevalence of four TTIs among blood donors on Bioko Island, Equatorial Guinea (EG).

**Methods:**

A retrospective survey of blood donors from January 2011 to April 2013 was conducted to assess the presence of HIV, HBV, HCV and *T*. *pallidum*. The medical records were analyzed to verify the seroprevalence of these TTIs among blood donations stratified by gender, age and geographical region.

**Results:**

Of the total 2937 consecutive blood donors, 1098 (37.39%) had a minimum of one TTI and 185 (6.29%) harbored co-infections. The general seroprevalence of HIV, HBV, HCV and *T*. *pallidum* were 7.83%, 10.01%, 3.71% and 21.51%, respectively. The most frequent TTI co-infections were HBV-*T*. *pallidum* 60 (2.04%) and HIV-*T*. *pallidum* 46 (1.57%). The seroprevalence of HIV, HBV, HCV and *T*. *pallidum* were highest among blood donors 38 to 47 years, 18 to 27 years and ≥ 48 years age, respectively (*P*<0.05). The seroprevalence of TTIs varied according to the population from which the blood was collected on Bioko Island.

**Conclusions:**

Our results firstly provide a comprehensive overview of TTIs among blood donors on Bioko Island. Strict screening of blood donors and improved hematological examinations using standard operating procedures are recommended.

## Introduction

Blood donation save millions of lives worldwide each year [1]. Although blood transfusion plays an essential role in the supportive care of medical patients, unsafe transfusion practices lead to the persistence of transfusion-transmissible infections (TTIs), including human immunodeficiency virus (HIV), hepatitis B and C virus (HBV and HCV, respectively), *Treponema pallidum*, arboviruses, malaria, filariasis and occasionally diseases such as toxoplasmosis and brucellosis [2,3]. According to the World Health Organization (WHO) recommendations, all blood donations should be screened for HIV, HBV, HCV and *T*. *pallidum*. Unsafe transfusion is incredibly expensive from both human and economic points of view. In the integrated strategy recommended by WHO, including selecting blood donors with a low risk for TTIs and effective laboratory monitoring is fundamental, and it has reduced the hazard of transmission to relatively low levels over the past two decades [2,4,5]. Nonetheless, in developing countries, a major proportion of donated blood presents risks as it is neither screened for all of the predominant TTIs nor is screening performed in a quality-controlled manner [6,7].

African countries require the greatest amount of blood transfusions worldwide while simultaneously facing tremendous suffering and pain from serious TTIs [1,3]. In fact, poor socio-economic factors, such as poverty, population growth, urbanization, and unstable environments, also directly or indirectly influence the transmission of TTIs [8]. The prevalence rates of TTIs in middle- and low-income African countries are much higher than that in high-income countries in Europe and North America. Risks for TTIs in Africa include a high HIV prevalence in the general populations and among blood donors and the lack of sufficient screening devices and skilled medical professionals to guarantee sustainable operations [6]. In countries with limited health care systems, rapid and simple serological tests are accepted by the WHO for use as TTI screening methods and are thus used in almost every hospital-based blood bank in Africa [9,10].

The prevalence of TTIs among blood donations can be used as a valuable indicator to assess the safety of the blood supply and the potential risk of infection. Changes in the prevalence may also reflect trends in the infections of interests among the general population. Previous epidemiological data of immigrants from Equatorial Guinea (EG) indicated an incredibly high prevalence of HIV, HBV, HCV and HDV infections in EG [8,11]. The current blood safety guidelines necessitate blood banks to routinely perform serological testing for HIV, HBV, HCV and *T*. *pallidum*. However, there are no comprehensive data about the overall seroprevalence of TTIs. Bioko is an island of EG, which lies approximately 100 km off the coast of southern Nigeria and 160 km northwest of continental EG. Malabo, the capital of EG, is located on Bioko Island. The purpose of the current study was to assess the seroprevalence of HIV, HBV, HCV, and *T*. *pallidum* among blood donors on Bioko Island, which facilitated a comprehensive perspective of TTIs in EG.

## Materials and Methods

### Study area and subjects

**Fig 1:**
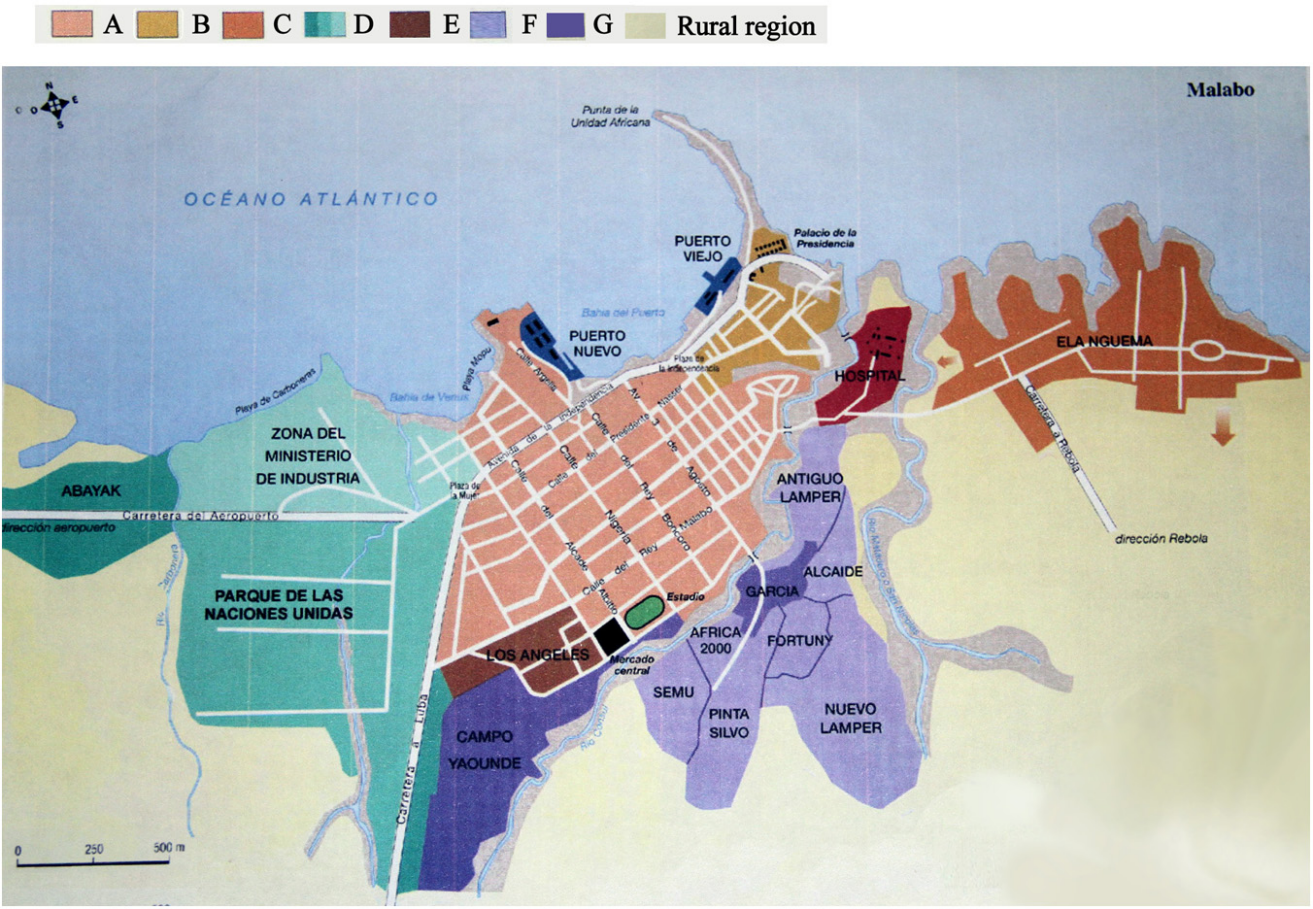
The geographic location of our research population. Malabo city was divided into eight regions including the old city center (A), the presidential office and residential area (B), the public residential area from previous planning (C), the modern residential area with a low population density (D), the modern residential area with a high population density (E), the un-planned residential area with a different population density (F) and the un-planned residential area with a high population density (G). The rural area included Luba, Moka, Riaba and Baney on Bioko Island.

The study regions are presented in Fig 1. The research area included two parts (Malabo City and Rural Region). According to an urban planning map of Malabo, the city is divided into eight regions including the old city center (A), the president office and the residential area (B), the public residential area from previous planning (C), the modern residential area with a low population density (D), the modern residential area with a high population density (E), the unplanned residential area with a different population density (F) and the unplanned residential area with a high population density (G). The rural areas of Bioko Island include Luba, Moka, Riaba and Baney.

A retrospective analysis of donor data from January 2011 to April 2013 at the Blood Bank of Malabo Region Hospital was conducted. All studies were approved by the Ethics Committee of Malabo Regional Hospital. The hospital is a major public hospital that provides health services to approximately 266,000 inhabitants (2001 census) on Bioko Island, EG. The voluntary donors were all healthy subjects selected after responding to a panel of questions, including questions about their medical histories. All donors answered the questions, which were designed to exclude donors who (a) had a recent ill history or received a blood transfusion more than once; (b) were less than 18 years or greater than 60 years old; (d) had a body weight less than 50 kilogram; (e) had a hemoglobin value less than 12.5 g/dL; or (f) had donated blood within 3 months. The socio-demographic characteristics of selected donors were recorded in a database. However, according to the limitation of the survey (a retrospective analysis of blood donor medical records), informed consent was not acquired from the study participants. Thus, the patient information was anonymous and de-identified prior to analysis. The seroprevalence rates of HIV, HBV, HCV, and *T*. *pallidum* were output in percentages and expressed with 95% confidential intervals (95% CI), which were calculated with a confidence interval calculator for proportions [12]. Pearson’s χ^2^ tests or Fisher’s exact tests (for values lower than 5) were used to assess categorical data.

### Sample collection

Blood samples were collected from each subject into an un-anticoagulated and an EDTA-K_2_ anticoagulated tube. The un-anticoagulated blood samples were allowed to clot naturally at room temperature and were then centrifuged at 2,000 rpm for 10 min to separate the serum.

### Serology

The serum samples were assessed for antibodies to HIV types 1 and 2, hepatitis B surface antigen (HBsAg), HCV and *T*. *pallidum* by colloidal gold immunochromatographic assay test strips (HIV and HbsAg: Alere^TM^, China; HCV and *T*. *pallidum*: Good Biotech Corp, Taiwan, China; EY Laboratories Inc., USA). The results from all samples that were reactive to HIV were confirmed by enzyme-linked immunosorbent assay (ELISA) kits (Murex HIV 1.2.0, Abbott). All samples positive for HBV, HCV and *T*. *pallidum* were confirmed with identical test kits. A result was considered positive only when both results were positive.

### Statistical analysis

**Fig 2:**
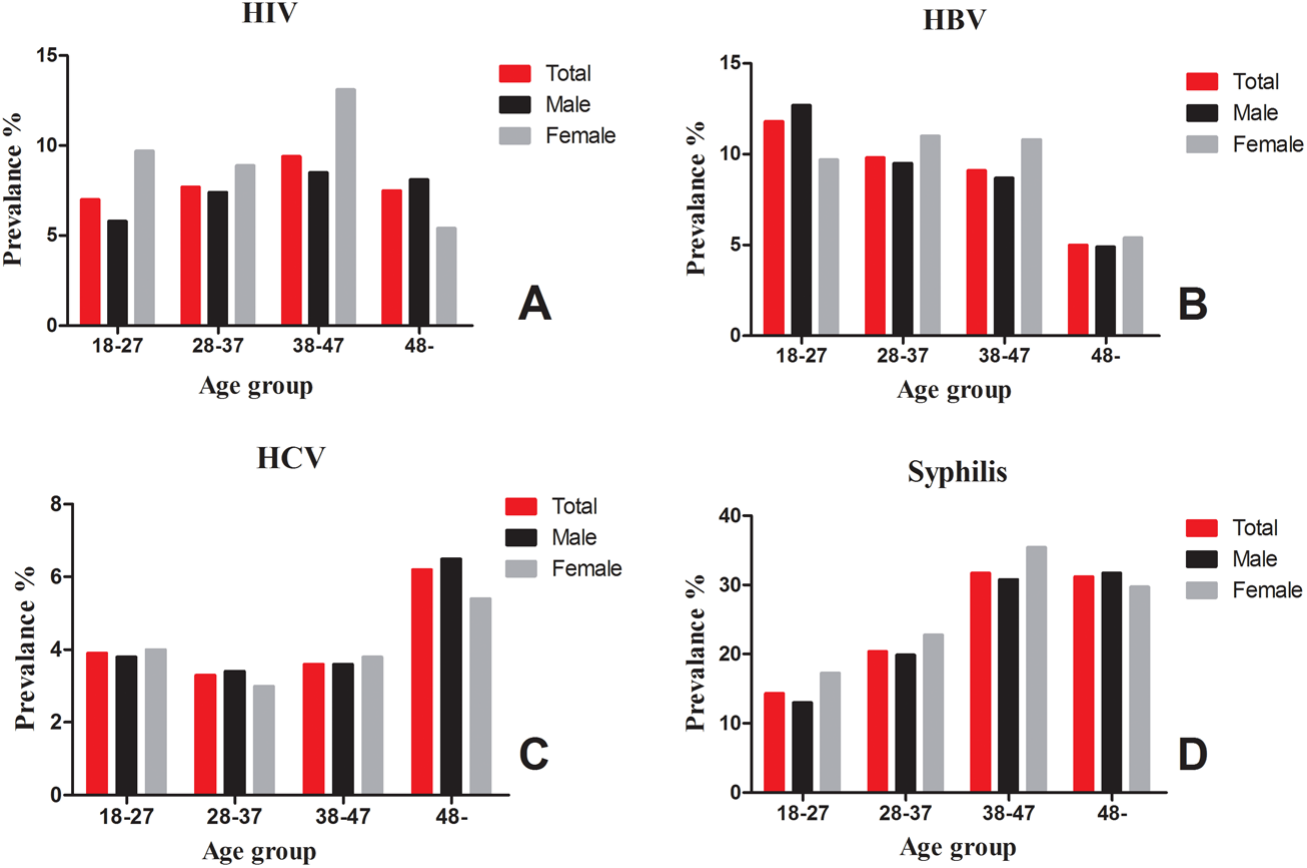
The distribution of the TTI seroprevalence rates among the blood donors.

The data were analyzed by the statistical package for social science for Windows version 17.0 (SPSS Inc., Chicago, IL, USA). The results were stratified by gender, age and the geographical region from which the donations came (Figs 1 and 2). Statistical significance was defined as a *P* < 0.05.

## Results

### The demographic characteristics of the blood donors

As shown in Table 1, a total of 2,937 blood donors were screened at the blood bank of Malabo Regional Hospital from January 2011 to April 2013. Almost half (41.13%) of the donors were 28–37 years old, and the average age of the study subjects was 32.57 ± 8.56 (mean ± SD, years). Of these blood donors, 2,256 (76.81%) are male and 681(23.19%) are female, producing a male/female ratio of 3:1. Most of the male blood donors (43.04%, 971/2,256) were 28–37 years old, whereas most of the female blood donors (40.67%, 277/2,937) were 18–27 years old.

**Table 1:** The socio-demographic characteristics of blood donors from the Malabo Regional Hospital on Bioko Island, Equatorial Guinea, 2011–2013.

Characteristics	Number (%)
**Age group (years)**	
18–27	932(31.73)
28–37	1,208(41.13)
38–47	637(21.69)
≥48	160(5.45)
**Gender**	
Male	2,256(76.81)
Female	681(23.19)
**HIV**	
Positive	230(7.83)
Negative	2,707(92.17)
**HBV**	
Positive	294(10.01)
Negative	2,643(89.99)
**HCV**	
Positive	109(3.71)
Negative	2,828(96.29)
***Treponema pallidum***	
Positive	632(21.52)
Negative	2,305(78.48)

### The seroprevalence of HIV, HBV, HCV and *Treponema pallidum*

The seroprevalence rates of HIV, HBV, HCV and *T*. *pallidum* were 230 (7.83%, 95% CI: 6.85–8.80), 294 (10.01%, 95% CI: 8.92–11.10), 109 (3.71%, 95% CI: 3.03–4.39) and 632 (21.51%, 95% CI: 20.02–23.00), respectively (Table 1). Of all the donated blood, 1,098 (37.39%, 95% CI: 35.64–39.14) specimens harbored at least one of TTIs and 185 (6.29%, 95% CI: 5.41–7.17) were infected with multiple pathogens. Detailed information about the co-infections is presented in Table 2II. For multiple infections, the most frequent combinations were HBV-*T*. *pallidum* (32.43%, 60/185) and HIV- *T*. *pallidum* (24.86%, 46/185).

**Table 2:** The co-infection seroprevalence of HIV, HBV, HCV and *Treponema pallidum* among blood donors on Bioko Island, Equatorial Guinea, 2011–2013.

Co-infections	Number (%)	Frequency (%, 95% CI)
HIV-HBV	29 (15.67)	(0.99, 0.63–1.35)
HIV-HCV	8 (4.32)	(0.27, 0.08–4.58)
HIV-*T*. *pallidum*	46 (24.86)	(1.57, 1.94–3.66)
HBV-HCV	8 (4.32)	(0.27, 0.08–4.58)
HBV-*T*. *pallidum*	60 (32.43)	(2.04, 1.53–2.55)
HCV-*T*. *pallidum*	26 (14.05)	(0.89, 0.53–1.21)
HIV-HBV-*T*. *pallidum*	5 (2.70)	(0.17, 0.02–0.32)
HBV-HCV-*T*. *pallidum*	3 (1.62)	(0.10, 0.02–0.30)
Total	185 (100)	(6.30, 5.42–7.18)

As shown in Fig 2, the overall seroprevalence of these four pathogens varied by the donors’ age and gender. At the blood bank in the Malabo regional hospital, donors 38–47 years old displayed a significantly higher positivity rate for HIV and *T*. *pallidum* (HIV: 9.4%, 95% CI: 7.13–11.67; *T*. *pallidum*: 31.7%, 95% CI: 23.48- 39.92) (Fig 2A and 2D). The seroprevalence of HBV was about two-fold higher in the youngest age group compared with the oldest age group (Fig 2B). In contrast to HBV, the seroprevalence of HCV peaked in the age group ≥ 48 years (6.2%, 95% CI: 2.46–9.94) (Fig 2C). All of the seroprevalence rates were higher in females than males. However, only the HIV seroprevalence and *T*. *pallidum* seroprevalence were statistically significantly different in women compared with males (*P* < 0.05). Because females might have been excluded as blood donors at a greater frequency than males due to lower weight or hemoglobin levels, further investigation of this difference should be performed.

### Seroprevalence in different geographical regions

As shown in Table 3, the seroprevalence of HIV, HBV, HCV and *T*. *pallidum* presented by geographical regions. Among the study population, 2,443 (83.18%) and 321 (10.71%) of donors came from Malabo city and the Rural Region, respectively. On Bioko Island, infection with *T*. *pallidum* was the most prevalent TTI in every geographical region, while HCV infection was the least prevalent. The overall seroprevalence of each TTI in Malabo City was little higher than that in Rural Region. However, there was no significant difference between them (*P*>0.05).

**Table 3:** The seroprevalence of HIV, HBV, HCV and *Treponema pallidum* in different regions with diverse populations on Bioko Island, Equatorial Guinea, 2011–2013.

Region	Population	HIV		HBV		HCV		*T*. *pallidum*	
		No.	%, 95% CI	No.	%, 95% CI	No.	%, 95% CI	No.	%, 95% CI
Malabo city	2 443	200	8.18 (7.09–9.27)	255	10.43 (9.22–11.64)	94	3.84(3.08–4.60)	528	21.61 (19.98–23.24)
A	80	4	5.00 (0.22–9.78)	4	5.00(0.22–9.78)	5	6.25(0.95–11.55)	17	21.25(12.29–30.21)
B	34	2	5.88 (0.00–13.78)	2	5.88(0.00–13.78)	1	2.94(0.00–8.62)	6	17.64(4.83–30.35)
C	591	45	7.61 (5.47–9.75)	61	10.32(7.87–12.77)	25	4.23(2.61–5.85)	102	17.25(14.20–20.30)
D	390	30	7.69 (5.05–10.33)	30	7.69(5.05–10.33)	9	2.30(0.81–3.79)	75	19.23(15.32–23.14)
E	129	9	6.97 (2.58–11.36)	19	14.72(8.61–20.83)	6	4.65(1.02–8.23)	22	17.05(10.56–23.54)
F	851	100	11.75(9.59–13.91)	108	12.69(10.45–14.93)	38	4.46(3.07–5.85)	248	29.14(26.07–32.19)
G	368	10	2.71(1.05–4.37)	31	8.42(5.57–11.25)	10	2.71(1.05–4.37)	58	15.76(12.04–19.48)
Rural Region	321	24	7.47(4.59–10.35)	24	7.47(4.59–10.35)	7	2.18(0.58–3.78)	64	19.93(15.56–24.30)
Unknown	173	6	3.46(0.74–6.18)	15	8.67(4.48–12.86)	8	4.62(1.49–7.75)	40	23.12(16.84–29.40)
Total	2 937	230	7.83 (6.85–8.80)	294	10.01 (8.92–11.10)	109	3.71 (3.03–4.39)	632	21.51(20.02–23.00)

**Note:** A, B, C, D, E, F and G represent the old city center, the presidential office and the residential area, the public residential area from previous planning, the modern residential area with a low population density, the modern residential area with a high population density, the un-planned residential area with a different population density and the un-planned residential area with a high population density, respectively. The rural area included Luba, Moka, Riaba and Baney on Bioko Island.

Among the different regions of Malabo City, our finding indicated that the un-planned residential area with a different population density (F) had the highest frequency of donations testing positive for HIV, *T*. *pallidum* and HBV, while the un-planned residential area with a high population density (G) had the lowest seroprevalence of HIV and *T*. *pallidum*. Chi-square tests for the various infection markers revealed that the seroprevalence of HIV and *T*. *pallidum* was significantly different between the F region and the average of Malabo City (*P*<0.05). The old city center (A) had the highest seroprevalence of HCV in the eight regions of Malabo City, but there was no significant difference among them (*P*>0.05).

## Discussion

In this study, we found a high prevalence rate of HIV infection (7.83%) among blood donors on Bioko Island, EG. Our results are consistent with the observed increasing seroprevalence of HIV in the general population in EG [8,11]. The prevalence of HIV increased from 1.1% in 1989–1992 to 3.48% in 1997, to 3.2% in 2004 and to 17.8% in 2011 [8,11]. The current seroprevalence of HIV is lower the in general population in EG and immigrants from EG living in Spain (2004: HIV, 10.8%) but is much higher among the rural population (1999: HIV, 0.6%) in EG [8,11]. Compared with other African countries, the overall seroprevalence of HIV in this study is higher than Nigeria (3.1%) [13], Burkina Faso (1.8%) [14], Cameroon (1.8%) [15] and Ethiopia (4.5%) [13] but is lower than in Tanzania (8.7%)[16]. Consistent with previous reports [17,18], the seroprevalence of HIV is significantly different between females and males (*P*<0.05), and further investigation is urgently needed to explain this important finding.

As one of most important TTIs, HBV infection is common in various African populations with a seroprevalence ranging from 0.2% to 20% [17, 18]. Our study found a 10.01% positivity rate for HBsAg among the donor population on Bioko Island. This prevalence value is lower than that of the neighboring countries including Cameroon (14.1% to 3 1%) and Gabon (24%) [19,20]. It is worth noting that HBsAg positivity is not limited to any particular age group, and a higher proportion of HBV was observed in donors aged 18 to 27 years. This finding is similar to Basaras’s report of blood donors in rural areas of EG [4]. They also found a higher seroprevalence of HBsAg among donors aged less than or equal to 18 years (13.4%) [4]. Such a higher seroprevalence among younger donors might be explained by the impact of mother-to-child vertical transmission, and further investigation is needed to support our hypothesis. Unfortunately, because of the poor infrastructure of the health system, we could not obtain enough information from the birth records at the Malabo regional hospital to support this viewpoint.

HCV seroprevalence among our donors is 3.71%, which is lower than values reported from Nigeria (6.0%) [6], Burkina Faso (6.3%) [14], Ethiopia (5.8%) [13], and Tanzania (8%) [16] but is higher than values reported from Cameroon (1.65%) [15]. Compared to a previous report [4], the prevalence of HCV increased from 1.7% in 1999 to 3.71% currently on the island. Our study also highlights that the predominantly high seroprevalence of *T*. *pallidum* antibodies may indicate a high-density carrier state or an active infection in the population on the island, which is higher than many previous reported prevalence rates in Africa [6,13,14,16]. The 24.86% HIV-*T*. *pallidum* co-infection rate in this study is higher than that observed in the neighboring country of Nigeria (5.6%) [21].

The high prevalence rates require attention and effective measures to ensure safe blood transfusion. We found that the seroprevalence of TTIs in un-planned residential areas with a different population density is significant higher than that of other areas of the island. This leads us to encourage the local government to take measures to control the spread of TTIs in this area. Further studies of the TTI status of the general population are necessary to determine the population prevalence on Bioko Island, EG.

In conclusion, our study indicates a remarkably high prevalence of TTIs on Bioko Island, and thus strict screening of blood donors and strengthening of hematological examinations, using standard operating procedures, are unquestionably recommended.
